# For better or worse: Factors predicting outcomes of family care of older people over a one-year period. A six-country European study

**DOI:** 10.1371/journal.pone.0195294

**Published:** 2018-04-03

**Authors:** Daniel Lüdecke, Barbara Bien, Kevin McKee, Barbro Krevers, Elizabeth Mestheneos, Mirko Di Rosa, Olaf von dem Knesebeck, Christopher Kofahl

**Affiliations:** 1 University Medical Center Hamburg-Eppendorf, Department of Medical Sociology, Martinistr. Hamburg, Germany; 2 Medical University of Bialystok, Department of Geriatrics, Bialystok, Poland; 3 Dalarna University, School of Education, Health and Social Studies, Falun, Sweden; 4 Linköping University, Department of Medical and Health Science, Division of Health Care Analysis, Linköping, Sweden; 5 50+ Hellas, Ammohostou 6, Halandri Attiki, Greece; 6 National Institute of Health and Science on Aging, Ancona, Italy; University of Heidelberg, Germany, GERMANY

## Abstract

**Objectives:**

Demographic change has led to an increase of older people in need of long-term care in nearly all European countries. Informal carers primarily provide the care and support needed by dependent people. The supply and willingness of individuals to act as carers are critical to sustain informal care resources as part of the home health care provision. This paper describes a longitudinal study of informal care in six European countries and reports analyses that determine those factors predicting the outcomes of family care over a one-year period.

**Methods:**

Analyses are based on data from the EUROFAMCARE project, a longitudinal survey study of family carers of older people with baseline data collection in 2004 and follow-up data collection a year later in six European countries (Germany, Greece, Italy, Poland, Sweden, and the United Kingdom), N = 3,348. Descriptive statistics of the sample characteristics are reported. Binary logistic random-intercept regressions were computed, predicting the outcome of change of the care dyad’s status at follow-up.

**Results:**

Where care is provided by a more distant family member or by a friend or neighbour, the care-recipient is significantly more likely to be cared for by someone else (OR 1.62) or to be in residential care (OR 3.37) after one year. The same holds true if the care-recipient has memory problems with a dementia diagnosis (OR 1.79/OR 1.84). Higher dependency (OR 1.22) and behavioural problems (OR 1.76) in the care-recipient also lead to a change of care dyad status. Country of residence explained a relatively small amount of variance (8%) in whether a care-recipient was cared for by someone else after one year, but explained a substantial amount of variance (52%) in whether a care-recipient was in residential care. Particularly in Sweden, care-recipients are much more likely to be cared for by another family or professional carer or to be in residential care, whereas in Greece the status of the care dyad is much less likely to change.

**Discussion:**

The majority of family carers continued to provide care to their respective older relatives over a one-year period, despite often high levels of functional, cognitive and behavioural problems in the care-recipient. Those family carers could benefit most from appropriate support. The carer/care-recipient relationship plays an important role in whether or not a family care dyad remains intact over a one-year period. The support of health and social care services should be particularly targeted toward those care dyads where there is no partner or spouse acting as carer, or no extended family network that might absorb the caring role when required. Distant relatives, friends or acquaintances who are acting as carers might need substantial intervention if their caregiving role is to be maintained.

## Introduction

In nearly all European countries, demographic developments have led to both a relative and absolute increase in the proportion of older adults in the population. Increasing longevity and an aging population mean an increase in the number of older citizens in need of long-term care and the length of time between when care is initially needed and death. The care and support needed by dependent people is primarily provided in the community by ‘informal’ networks of relatives or friends and close acquaintances (hereafter referred to as ‘family’ care) and supplemented by the ‘formal’ care provided by health and social services [1–3]. Indeed, the economic value of such family care often exceeds the cumulative costs for home care services and nursing homes in many European countries [4,5]. Governments therefore should have an interest in supporting informal care as this covers a substantial proportion of potential health care costs. However, human resources for informal care are fading. Demographic factors and socioeconomic changes, such as a decline in intergenerational cohabitation in smaller families and an increasing proportion of women entering the labour markets mean that the potential for family care is fading. Not only, but also for this reason, expenditures for the ambulatory health care sector have increased during the past decade across Europe [6–9]. Service provision for and legal recognition of family carers, however, vary between countries. Northern European countries have a high level of public service provision, while in liberal countries like the United Kingdom (UK), Ireland and the Netherlands the focus lies more on recognising and valuing family carers as a group of citizens with special rights. In southern and eastern European countries, there is a relatively low level of residential and home care services, with family carers providing more care with hardly any professional support [1,10]. This paper describes a longitudinal study of informal care in six European countries and reports analyses that determine those factors predicting the outcomes of family care over a one-year period.

The supply and willingness of individuals to act as carers are critical to sustain informal care resources as part of the home health care provision. Unfortunately, however, family carers often prioritise the needs of their relatives as opposed to their own needs and, as a result, often put off or fail to make use of health promotion, prevention, or relief services for their own well-being. As numerous studies have shown, family care work is often physically, mentally and emotionally demanding. Caring for older people, in particular those with cognitive impairments, places extraordinary demands on family carers and takes a significant toll on their health [11–15]. The physical and mental burden a family carer experiences can also be influenced by the nature of the relationship between carer and care-recipient. Intragenerational care, as provided by spouses, is typically associated with worse health outcomes for carers than intergenerational care, where children (in-law) or more distant relatives provide care. The stronger the personal commitment between family carer and care-recipient, and the identification on the carer’s part with the carer’s role, the more the carer risks experiencing high levels of burden as a result of providing care [16,17]. Strategies used by family carers to reduce the negative impact of care can include both seeking professional help and accessing more support from members of one’s informal networks. However, with a lack of formal and informal help and increasing dependency and need for care in the care-recipient, the burden of care can rise to a point when unwanted outcomes occur such as the early institutionalization of the care-recipient and elder abuse [18,19,20]. Only a few studies have compared the outcomes of care over time as they relate to different care dyad relationships, such as spouses versus other, mostly younger family carers or distant relatives or acquaintances.

Our hypothesis is, first, that family carers are more likely to give up care when the care-recipient is more severely dependent and/or suffers from higher severity of dementia and behavioural problems. Second, we assume that the relationship between family carer and care-recipient is associated with whether, after one year, the care-recipient is still living at home or being cared-for in a long-term care facility.

This paper considers three main research questions that relate to a gap in our understanding of those factors that predict the outcomes of family care of older people. First, what factors predict whether a family carer gives up personal care for an older person still living at home within one year? Second, what factors predict whether an older family care-recipient is still cared for by the same family carer after one year or is a resident in a long-term care facility? Third, is the outcome of family care influenced by the relationship between carer and care-recipient? A subsidiary research question is whether variance in the outcomes of family care of older people can be explained by country of residence.

## Methods

### 2.1 Sampling and participants

Analyses are based on data from the EUROFAMCARE (EFC) project, a longitudinal study with baseline data collection in 2004 and follow-up data collection a year later. At baseline in six European countries a total of 5,923 family carers (Greece (EL), n = 1,014; Italy (IT), n = 990; United Kingdom (UK), n = 995; Sweden (SE), n = 921, Poland (PL), n = 1,000; Germany (DE), n = 1,003) were interviewed at home using a common assessment tool (CAT) [21] that focused on their experiences and circumstances in providing care to an older relative. The original data collection aimed at ensuring a sample reflecting the variety of existing caregiving situations. As in most European countries, no representative lists of family carers are available, a non-random sampling strategy was pursued. Each country’s national territory was divided into at least three sample areas, which were then again subdivided into rural, urban, and metropolitan place of residence. In each of these selected sample zones, family carers were identified and recruited through a saturation method that took into account local conditions [22].

For the purposes of the study a family carer was defined as someone occupying the primary role in personally providing at least four hours of care or support per week to a relative, friend or neighbour aged 65 years or older. Potential participants solely providing financial support were excluded. No care-receivers were interviewed and so all data collected on care-receivers is based upon information provided by their respective carers. Before data collection began, the CAT-questionnaire was tested in two cross-national pilot studies. A standard evaluation protocol was agreed upon by the EFC consortium to ensure comparable national samples reflecting the carers' universe by providing common guidelines on sample unit (i.e., definition of family carer of older people), sampling and recruitment strategies, and standardized training of interviewers for the administration of the CAT-questionnaires in a face-to-face setting [22]. An attempt was made to contact all baseline participants at follow up one year later (2005). The response rate for the follow-up survey was approximately 57% (3,367 family carers). Response rates varied between countries from 28% in Greece to 88% in Italy and Poland. The sample in the present study is based on the follow-up survey of the EFC sample, but only includes those family carers who provided care to an older person living in the community at baseline. Older persons in residential care at baseline were excluded from the analyses. The final sample therefore consists of 3,348 family carers (EL: n = 281; IT: n = 860; UK: n = 318; SE: n = 568, PL: n = 875; DE: n = 446, see Table 1).

**Table 1:** Sample size per country at baseline and follow-up, and final sample size.

*Country*	*N at Baseline*	*N at follow-up (Response Rate)*	N for cleaned follow-up (final sample)*
Greece (EL)	1,014	282 (27.8 %)	281
Italy (IT)	990	863 (87.2 %)	860
UK	995	320 (32.2 %)	318
Sweden (SE)	921	575 (62.4 %)	568
Poland (PL)	1,000	875 (87.5 %)	875
Germany (DE)	1,003	452 (45.1 %)	446

* In the final sample, family carers of older persons *in residential care at baseline* were excluded.

National ethical committees approved the study in all countries. In Germany, the project has been approved by four ethical committees from four different German states (Landesärztekammer Baden-Württemberg, Ärztekammer Nordrhein, Ärztekammer Schleswig-Holstein and Ärztekammer Sachsen-Anhalt) on behalf of the federal states where the carer’s interviews have been conducted. The study did not require approval from the Greek national or local ethics committee. The Legal Representative of the National School of Public Health, Athens, was consulted regarding the study and gave the advice that there were no ethical problems in administering the questionnaire as participation was voluntary and all potential subjects were informed in writing of their right not to participate or to refuse to answer certain questions if they wished, without any adverse consequences. In Italy, the project was approved by the institute’s ethical committee, Comitato di Bioetica INRCA, Istituto Nazionale di Riposo e Cura per Anziani, Ancona (National Institute of Health and Science on Aging, Bioethics Advisory Committee). Poland received permission from the Ethical Commission of Medical Academy of Bialystok, including the permission of the General Inspector for Protection of Privacy of Personal Data in Poland. For Sweden, the ethical committee at Linköpings universitet approved the study. In the UK, approval for the study was provided by the Multi Research Ethics Committee (MREC), a national body for multi-centre studies.

### 2.2 Baseline data (independent variables)

A wide range of variables were measured in the CAT, the choice of which was theoretically driven, aimed at giving a holistic picture of the care setting at home. This includes the specific characteristics of the carer (*gender*, *age*, *educational attainment* and *religiousness*), the degree of dependency and amount of care needed by the care-recipient (*dependency*, *memory problems* and *behavioural problems*), as well as the particular characteristics of the care situation itself (*hours of care per week*, *unmet needs*, *negative impact of care*, *positive value of care* and *carer/care-recipient relationship*).

#### Carer’s characteristics

The *age* and *gender* of the carer was recorded. To assess carers’ *educational attainment*, some countries used an open response question while other countries used lists of national educational categories. A synthesis was achieved at the European level by recoding the country-specific categories into three levels of educational attainment: ‘low’, ‘intermediate’ and ‘high’. A measure of religiousness was provided by a question asking carers how religious they considered themselves, with response options “not religious at all”, “quite religious” and “very religious”.

#### Care-recipient’s needs and limitations

For our measure of care-recipient *dependency*, the carer indicated the older care-receiver’s functional limitation on a four-point scale: ‘independent’; ‘slightly dependent’; ‘moderately dependent’ or ‘severely dependent’. *Memory problems* were assessed by asking the carer if the care-recipient had any memory problems, and if so, whether a physician has given a diagnosis, resulting in a variable with three response categories: ‘No memory problems’, ‘memory problems, but no dementia diagnosis’ and ‘memory problems with dementia diagnosis’ (Alzheimer’s Disease and other types of dementia). *Behavioural problems* were measured by the behavioural component of BISID (Behavioural and Instrumental Stressors in Dementia; [23]). The three items ask how often the care-recipient 1) wanders around or behaves dangerously, 2) has difficulties holding normal conversations and 3) behaves in ways the carer finds upsetting; response options are ‘never’ (0), ‘rarely’ (1), ‘sometimes’ (2) and ‘most of the time’ (3). Item scores were added and median-dichotomized into ‘no or few behavioural problems’ (scores 0 to 2) or ‘behavioural problems’ (scores 3 to 9).

#### Characteristics of the care situation

*Hours of care per week* provided by the carer was assessed by an open-ended question. The care-recipient’s *unmet needs* were measured by asking carers whether or not the care-recipient required help in each of a series of need domains (health; physical/personal; mobility; emotional/psychological/social; domestic; financial management; financial support; and organising and managing care and support) and if so whether they would like to have more help to meet the care-recipient’s need. A ‘yes’ response to both components of the question indicated an unmet need in that specific need domain, with domains summed up to provide an overall score for unmet need. The *negative impact of care* as well as the *positive value of care* were measured by the COPE Index [24].

### 2.3 Outcomes (dependent variables)

Our outcome variable was a three-category representation of the *care dyad’s status at follow-up*. The care-recipient remaining in the community and being cared for by the same carer after one year represented ‘Unchanged status’. From the total sample this constituted n = 2,706. ‘Changed status—different carer’ was represented by the care-recipient remaining in the community but being cared for by a *different* family or even professional carer. From the total sample this constituted n = 407. ‘Changed status—in residential care’ was represented by the care-recipient no longer receiving care from a family carer but being in residential care. From the total sample this constituted n = 235. Where a care-recipient had died between baseline and follow-up, his/her care status at time of death was determined and the care-recipient allocated to one of the three status categories.

### 2.4 Statistical analyses

Three regression models were developed, each with a binary outcome, which was recoded based on the three-category variable *care dyad’s status at follow-up*. In Model 1, the binary outcome was ‘Unchanged status’ versus ‘Changed status—different carer.’ In Model 2, the binary outcome was ‘Unchanged status’ versus ‘Changed status—in residential care.’ Model 3 was the same as Model 1, but introduced an interaction term, *hours of care per week* x *carer/care-recipient relationship*. This third model was developed post-hoc of the first model to further investigate the profile of significant predictors. Using the *R statistics software* [25], binary logistic random-intercept regressions (generalized linear mixed effects models) were computed using the *lme4* package [26]. It allows for calculating (logistic) regression models while controlling random effects, i.e. to account for variation in the outcome variable for different clusters or groups. In this particular case, a *country of residence* variable was created and selected as random intercept (grouping level). Continuous control variables with very different scales of magnitude were centred and standardized to avoid problems with model convergence.

Response propensity weights were calculated to account for sample attrition between baseline and follow-up [27]. We chose a multilevel logistic regression model including the same predictors as auxiliary variables used in our main regression models to predict the propensity scores on respondents versus non-respondents. To reduce bias in the response propensities, “smoothing” via natural splines was applied to auxiliary variables where appropriate. Including country as a random effect accounts for the variability in response patterns by countries [28]. The adjusted inverse probability of response propensities was used as a weighting factor in our regression models. We found no large weight values (“outliers”), so trimming the weights was not necessary.

To analyse country specific effects on the outcome, the fixed and random effects of the mixed models are described separately. Conditional modes (which maximize the density of the random effects conditional on the variance-covariance parameters and the data) for the random effects are reported. Furthermore, we computed the intraclass correlation coefficient (ICC) to explore the proportion of variance due to the variable *country of residence*. Since random intercept logistic regression models have an unknown variance at level 1, the residual variances to calculate the ICC is fixed at $\frac{\pi^{2}}{3}$ [29].

Odds ratios (OR) are reported for the first two models. Wald tests were used to compute approximate p-values for the fixed effects estimates. Predicted probabilities were calculated for the interaction term of the third model. Figures and tables for regression models (including summary statistics) were created with the *sjPlot* package [30].

## Results

### 3.1 Descriptive results

The mean age of carers in our sample is 56 (SD ±14) years. The majority of them are females (78%). The care-recipients are on average 81 years old, with 69% of them being female. About 18% of carers have a high educational attainment. In Sweden, we find 26% of carers with high attainment, by comparison in the UK only 12% of carers have high educational attainment. Religiousness was more prevalent in the Southern European countries: 44% of family carers in Greece and 21% in Italy considered themselves as very religious. For Sweden and Germany, we have the lowest proportions (5%) of people saying they are very religious.

Looking at the care-recipient’s care needs and limitations, overall 67% are moderately to severely dependent, this figure as low as 51% in Poland and as high as 81% in Germany. Regarding memory problems, more than half of all care-recipients have some kind of memory problems, either with or without diagnosed dementia. This number is particularly high in Germany, but also in the UK and Sweden. Behavioural problems occur in one third of all care-recipients.

Concerning the care situation, half of all care-recipients receive more than 20 hours care per week. 32% of care-recipient have unmet needs in at least three need domains. However, there is considerable variation across countries in unmet need, with the figure for unmet need in at least three need domains as low as 14% in Sweden and 21% in the UK, while as high as 47% in Italy and 59% in Greece. In the majority of care dyads, the partner, child, or child in-law provides care for the care-recipient, with only 14% of carers being more distant relatives, neighbours or close friends. However, in Italy, Poland and the UK, this latter group of carers is more common (see Table 2).

**Table 2:** Carer characteristics, care-recipient care needs and limitations, and characteristics of the care situation by country and overall sample (N = 3.348, in%).

Variable	Country						Total
	EL	IT	UK	SE	PL	DE	
*Characteristics of carer*							
Female	87	78	83	73	77	78	78
High educational attainment	19	16	12	26	15	21	18
Very religious	44	21	12	5	10	5	14
*Care-recipient’s needs and limitations*							
Moderate to severe dependency	73	66	77	73	51	81	67
Memory problems (w/o dementia diagnosis)	19	25	37	32	19	26	25
Memory problems (dementia diagnosis)	23	24	20	20	23	39	24
Behavioural problems	40	37	30	40	28	46	36
*Characteristics of the care situation*							
More than 20 hours of care per week	69	53	58	37	56	54	53
High unmet needs^1^	59	47	21	14	24	35	32
High negative impact of care^2^	76	49	47	49	22	53	44
High positive value of care^2^	43	32	38	49	49	24	40
Carer relationship to care-recipient other than partner or child (in-law)	11	18	18	7	17	11	14
*Sample size per country (N)*	281	860	318	568	875	446	3,348

^1^ unmet needs in at least three need domains

^2^ median-split

### 3.2 Results for mixed effects models

The results of the logistic multilevel models, Model 1 (‘Unchanged status vs. Changed status—different carer’) and Model 2 (‘Unchanged status/Changed status—in residential care’) are presented in Table 3.

**Table 3:** Odds ratios (confidence intervals) and wald-p-values for variables predicting status of care dyad at follow-up: Changed status—different carer (Model 1, n = 3113) and changed status—in residential care (Model 2, n = 2941).

	Model 1: Changed status–different carer		Model 2: Changed status–in residential care	
	*OR (CI)*	*p*	*OR (CI)*	*p*
**Fixed Effects**				
*Characteristics of carer*				
Carer’s gender (female)	1.19 (0.90 – 1.59)	.229	1.33 (0.89 – 2.00)	.160
Carer's age*	0.99 (0.84 – 1.16)	.896	1.02 (0.80 – 1.30)	.878
Carer’s educational attainment (intermediate)^1^	1.26 (0.92 – 1.72)	.146	0.97 (0.65 – 1.44)	.878
Carer’s educational attainment (high)^1^	0.99 (0.67 – 1.47)	.969	1.01 (0.62 – 1.65)	.959
Carer’s religiousness (quite religious)^2^	0.61 (0.46 – 0.79)	**< .001**	1.16 (0.81 – 1.66)	.413
Carer’s religiousness (very religious)^2^	0.58 (0.38 – 0.88)	**.011**	1.81 (1.00 – 3.28)	**.049**
*Care-recipient’s care needs and limitations*				
Dependency	1.22 (1.05 – 1.41)	**.009**	1.24 (0.98 – 1.55)	.068
Memory problems (w/o dementia diagnosed)^3^	0.95 (0.69 – 1.30)	.738	1.14 (0.74 – 1.76)	.553
Memory problems (diagnosed dementia)^3^	1.79 (1.25 – 2.56)	**.001**	1.84 (1.13 – 3.00)	**.014**
Behavioral problems	1.31 (0.95 – 1.80)	.100	1.76 (1.15 – 2.68)	**.009**
*Characteristics of the care situation*				
Hours of care per week*	0.37 (0.29 – 0.46)	**< .001**	1.05 (0.88 – 1.26)	.575
Moderate unmet need^4^	1.10 (0.82 – 1.48)	.519	1.20 (0.81 – 1.76)	.369
High unmet need^4^	1.25 (0.92 – 1.69)	.161	1.43 (0.95 – 2.17)	.087
Negative Impact of care*	0.93 (0.79 – 1.10)	.405	1.04 (0.84 – 1.27)	.741
Positive Value of care*	1.00 (0.88 – 1.14)	.989	0.91 (0.77 – 1.09)	.307
Relationship to elder: children^5^	0.75 (0.50 – 1.13)	.176	1.98 (1.20 – 3.26)	**.008**
Relationship to elder: children in-law^5^	0.80 (0.48 – 1.35)	.408	1.71 (0.86 – 3.38)	.124
Relationship to elder: others^5^	1.62 (1.01 – 2.59)	**.046**	3.37 (1.78 – 6.39)	**< .001**
**Random Effects**				
N_country_	6		6	
ICC_country_	0.069		0.518	
Observations	2,707		2,567	
Deviance	1890.999		1072.977	

Reference categories

^1^ low educational attainment

^2^ not religious at all

^3^ no memory problems

^4^ no unmet needs

^5^ relationship to elder: spouses/partners

* Continuous variables were centred and standardized.

#### 3.2.1 Changed status—different carer (Model 1)

With regard to the sub-sample used in Model 1, at follow-up, approximately 87% of care dyads are unchanged, with the original carer remaining the primary carer of the care-recipient, while approximately 13% of carers no longer provide care to the care-recipient who remained in the community but is being cared for by a different family or professional carer.

In the model, religiousness is the only carer characteristic that is a significant predictor of change of status. Carers considering themselves as “quite” religious are less likely to give up care (OR 0.61). Odds for giving up care are even smaller for carers who consider themselves as “very religious” (OR 0.58). When considering the care-recipient’s needs and limitations, a higher level of dependency is associated with higher odds of change of status (OR 1.22), as is memory problems with dementia diagnosis (OR 1.79). Memory problems without dementia diagnosis, however, is not significant in the model.

Regarding characteristics of the care situation, a higher number of hours of care per week reduces the odds of a change of status (OR 0.37). Unmet need is not significant in the model, and this is the case also for negative impact and positive value of care. However, the carer/ care-recipient relationship is significant in the model. Compared to care provided by a spouse or partner, care provided by an ‘other’ family member (i.e., an uncle or aunt, a friend or neighbour) results in increased odds of a change of status (OR 1.62). Care provided by a child or a child-in-law is not significant.

**Fig 1:**
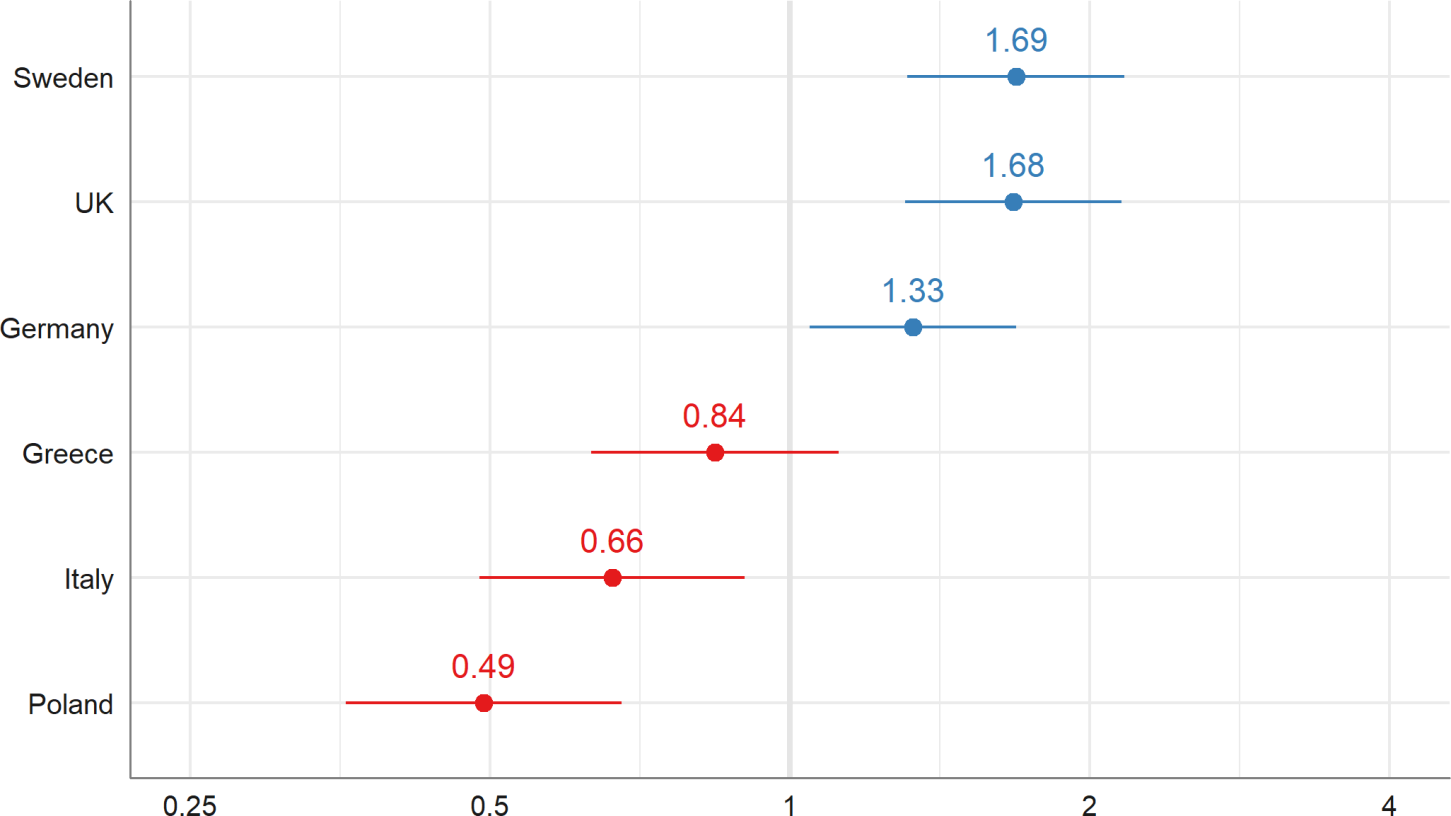
Variance in change of status–different carer between country of residence: Conditional modes (random intercepts, group levels) of model 1.

The ICC for Model 1 is 0.069 (see Table 3), indicating that approximately 7% of the variance in change of status can be attributed to country of residence. Looking at the conditional modes of the random effects of model 1, we see this variation in detail (see Fig 1). Particularly in Sweden and the UK, but also in Germany, a changed status—different carer is more likely when compared to Greece, Italy, and especially Poland.

#### 3.2.2 Changed status—in residential care (Model 2)

With regard to the sub-sample used in Model 2, at follow-up, approximately 92% of care dyads were unchanged, with the original carer remaining the primary carer of the care-recipient, while approximately 8% of carers no longer provide care to the care-recipient who by that time was in residential care.

In the model, religiousness is the only carer characteristic that is a significant predictor of change of status. Carers who consider themselves as ‘very religious’ are more likely to give up care (OR 1.81). When considering the care-recipient’s needs and limitations, memory problems with dementia diagnosis and behavioural problems both increase the odds of a change of status (OR 1.84 and OR 1.76 respectively).

Regarding characteristics of the care situation, the carer/care-recipient relationship is a significant predictor of change of status. Compared to care provided by a spouse or partner, care provided by a child (OR 1.98), child-in-law (OR 1.71) or a ‘other’ family carer (OR 3.37) all increase the odds of a change of status with the care-recipient being in residential care.

**Fig 2:**
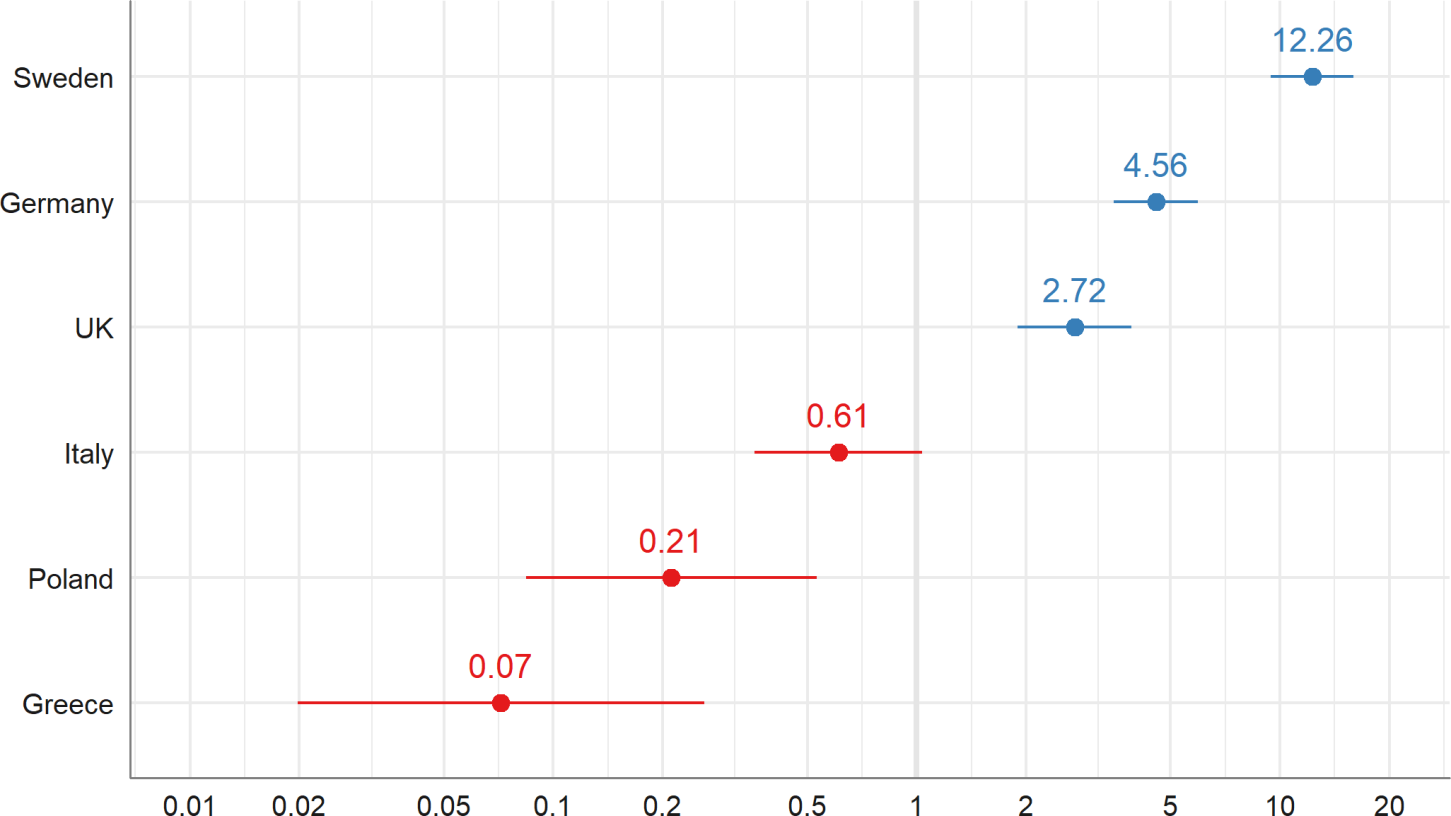
Variance in change of status–in residential care between country of residence: Conditional modes (random intercepts, group levels) of model 2.

The ICC for Model 2 is 0.518 indicating that about half (52%) of the variance in change of status—in residential care is due to country of residence. Looking at the conditional modes of the random effects of Model 2, we see large differences between the countries (see Fig 2). Especially in Sweden, but also in Germany and the UK, change of status—in residential care is more likely than in Italy, or, particularly, in Poland and Greece.

#### 3.2.3 Changed status—different carer: Further analysis with hours care per week x carer/care-recipient relationship interaction term (Model 3)

**Fig 3:**
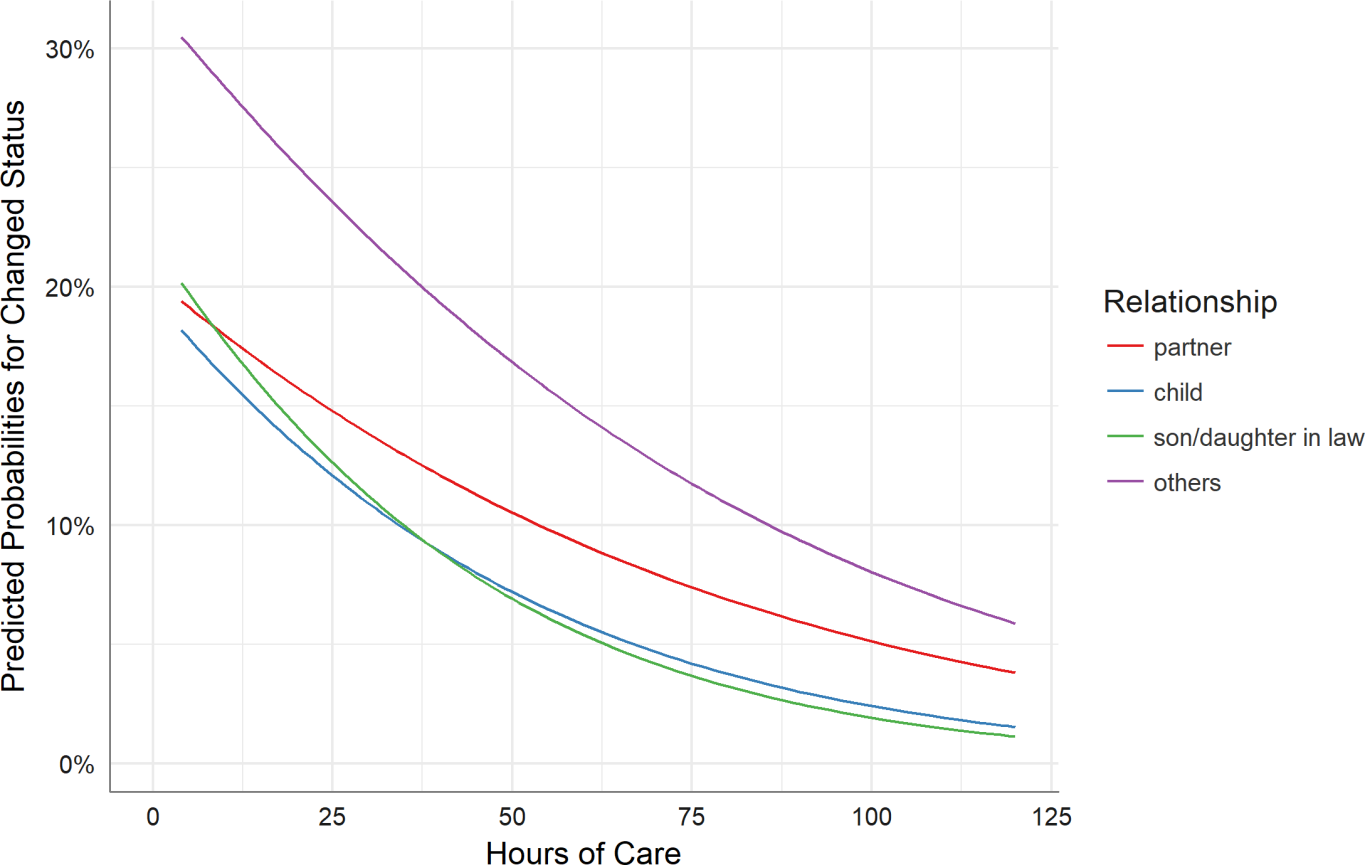
Predicted probabilities for change of status—Different carer, hours of care per week x carer/carer-recipient relationship interaction (Model 3).

In Model 1, the number of hours of care provided per week is a significant predictor of changed status—different carer, with a greater number of hours care per week associated with a reduction in the odds that the care-recipient will be cared for by a different carer. To further investigate this issue, another regression model was run, based on the first model but with an additional interaction term (hours of care per week x carer/care-recipient relationship). The results for Model 3 (see Table 4) indicate that the main effect for number of hours care per week is significant, while other main effects and the interaction terms are not. The effect of relationship on the association of hours of care per week and a change in status (different carer) is primarily present for fewer hours of care. As the amount of hours of care per week increase, differences in the carer/care-recipient relationship decrease. This can be seen in Fig 3, which shows the predicted probabilities for a change of status depending on hours of care per week and grouped by family care relationships. The odds of a change of status towards a different carer are lower for inter- or intragenerational care, and greater for ‘other’ family care relationships, especially when the hours of care per week are few.

**Table 4:** Odds ratios (confidence intervals) p-values for variables predicting a changed status (different carer) (Model 3).

	Model 3: Changed status–different carer	
	*OR (CI)*	*p*
**Fixed effects**		
*Characteristics of carer*		
Carer’s gender (female)	1.20 (0.90 – 1.59)	.222
Carer's age*	0.99 (0.85 – 1.16)	.925
Carer’s educational attainment (intermediate)^1^	1.25 (0.92 – 1.70)	.159
Carer’s educational attainment (high)^1^	0.98 (0.66 – 1.46)	.935
Carer’s religiousness (quite religious)^2^	0.61 (0.46 – 0.80)	**< .001**
Carer’s religiousness (very religious)^2^	0.58 (0.38 – 0.89)	**.012**
*Care-recipient’s care needs and limitations*		
Dependency	1.22 (1.05 – 1.42)	**.009**
Memory problems (w/o dementia diagnosed)^3^	0.95 (0.69 – 1.30)	.741
Memory problems (diagnosed dementia)^3^	1.81 (1.27 – 2.60)	**.001**
Behavioral problems	1.30 (0.94 – 1.79)	.109
*Characteristics of the care situation*		
Moderate unmet need^4^	1.09 (0.82 – 1.47)	.547
High unmet need^4^	1.24 (0.91 – 1.69)	.167
Negative Impact of care*	0.94 (0.80 – 1.11)	.495
Positive Value of care*	1.01 (0.88 – 1.15)	.919
Relationship to elder: children^5^	0.68 (0.43 – 1.07)	.095
Relationship to elder: children in-law^5^	0.66 (0.34 – 1.30)	.233
Relationship to elder: others^5^	1.73 (0.96 – 3.10)	.067
Hours of care per week*	0.44 (0.32 – 0.60)	**< .001**
*Interaction term*: Relationship status * Hours of care per Week		
Relationship to elder: children * Hours of care per week	0.68 (0.40 – 1.15)	.148
Relationship to elder: children in-law * Hours of care per week	0.56 (0.22 – 1.41)	.217
Relationship to elder: others * Hours of care per week	0.93 (0.44 – 2.00)	.862
**Random effects**		
N_country_	6	
ICC_country_	0.067	
Observations	2,707	
Deviance	1887.889	

Reference categories

^1^ low educational attainment

^2^ not religious at all

^3^ no memory problems

^4^ no unmet needs

^5^ relationship to elder: spouses/partners

* Continuous variables were centred and standardized.

## Discussion

The study reported in this paper sought to understand those factors that influence the outcomes of family care of older people. The longitudinal nature of the study, carried out via a standard protocol in six European countries, allowed us to determine over a one-year period those factors that predicted whether a family care dyad would be unchanged, or if the older care-recipient would be cared for by a different family or professional carer or be in residential care. We were particularly interested in the role of the relationship between carer and care-recipient in predicting these outcomes of family care. Additionally, we were able to explore the extent to which the care dyad’s country of residence explained variation in such outcomes.

Our main finding is that the carer/care-recipient relationship plays an important role in whether or not a family care dyad remains intact over a one-year period. When care is provided by a more distant family member or by a friend or neighbour–compared to care provided by a spouse or partner–a care-recipient is significantly more likely to be cared for by a different person than the one who provided care a year earlier, or to be in residential care. Furthermore, even where a child or child-in-law provides care, the care-recipient is more likely to be in residential care after a year than where care is provided by a spouse or partner. Thus, if one’s concern is in supporting and sustaining a family care dyad, our findings suggest that a relationship between carer and care-recipient other than spouse or partner increases the risk that the care dyad will not last in the longer term.

We interpret this finding in the light of other research that considers the motivations of family carers in providing care. While spouses often regard caring for their partner as a marital duty, children and children-in-law and, in particular, other relatives or acquaintances arguably commit to their caring role to a greater extent when they have more emotional closeness to the care-recipient or if they cope well with their role of carer. Hence, the association between care motives and the carer/care-recipient relationship is one explanation for a willingness to continue or preference for relinquishing care [13,31,32]. This is in line with other studies that show that family carers still continue to provide care despite heavy amounts of care needed, especially when there is a close emotional relationship between carer and care-recipient [17,33].

We found that the more hours of care dedicated to a care-recipient, the less likely a different carer will care for the care-recipient after one year. This finding appears counter-intuitive, and so was explored further by considering how hours of care provided interacts with the carer/carer-recipient relationship. Our analyses show that the association between the amount of hours of care per week and a change in status of the family care dyad is strongly influenced by the carer/carer-recipient relationship. A care-recipient is more likely to be cared for by a different person after one year when the care dyad is intergenerational, or particularly if a distant relative, friend or neighbour provides care. This finding holds true particularly when hours of care per week is low. Thus, the traditional marital promise “for better or worse” seems to hold true, in line with other studies that indicate the important role played by spouses in the care of older people [34].

A second key finding is that where the care-recipient has memory problems with a dementia diagnosis, that care-recipient is significantly more likely to be cared for by a different family member or professional carer than the one who provided care a year earlier, or to be in residential care. This finding is not so surprising given the number of studies that have demonstrated that caring for an older relative with dementia can be very demanding for the carer. It seems that there is a higher severity or a different quality in dementia-related memory problems compared to other memory problems. This can increase the level of burden experienced by the carer [12,35,36]. Our study also found that higher dependency and behavioural problems in the care-recipient lead to a change of care dyad status: high dependency predicted that the care-recipient would be with a different carer after a year, whereas behavioural problems predicted that the care-recipient is in residential care. Thus, there is a suggestion that functional limitations (dependency) can be a trigger for family care to be reallocated in some fashion within an informal care network [37], whereas behavioural problems, which are a frequent accompaniment to dementia, are sufficient to lead to family care ceasing altogether.

A third main finding from our analyses is that country of residence explained variance in the outcomes of family care. Country of residence explained a relatively small amount of variance in whether a care-recipient was cared for by a different carer after one year, but explained a substantial amount of variance in whether a care-recipient was in residential care after one year. Considering the extremes in our six countries, care-recipients in Sweden are much more likely to be cared for by another family or professional carer or to be in residential care after one year, whereas in Greece the status of the care dyad is much less likely to have changed after one year. This might be due to both the specific health care regime as well as normative characteristics in European countries. In Sweden, compared to other countries in our study, we find a well-developed structure of health care service provision [38]. The use of support services, including residential long-term care, seems much more accepted and the moral obligation to care for relatives as not as strong as in other countries [39]. The situation in Greece is the opposite in this regard, with hardly any support services available meaning there are few viable alternatives to family care, accompanied by a strong normative value system that sees family care as a ‘good thing’ [10,39]. In all, we can see a “gradient” from Scandinavian regimes (Sweden) over Bismarckian and liberal regimes (Germany and UK) to more family oriented regimes (Italy, Greece and Poland), in the likelihood that a family care dyad will change in status over a one year period. It is likely this gradient also reflects the different policy approaches in different countries reconciling paid work and care responsibilities [40]. Religious beliefs and moral obligations, which affect the situation of family care and which differ substantially between (especially Northern and Southern) European countries, may be further reasons for the obvious variation of the outcome in those countries. From our data, we have evidence for a gradient in religious attitudes that is associated with a change of status–different carer. Family carers who considered themselves as “quite religious” were more likely to continue personal care, while the odds were even higher for maintaining personal care for participants that are “very religious”. However, the opposite relationship was found with regard to change of status–in residential care, where there was a significant association between strong religious beliefs and a change of status. This finding is inconsistent with other research that mostly describes positive associations between religiousness and willingness to care [41,42]. One explanation might be that the perception of religiousness is associated with the possibility to attend church or to do spiritual activities. Carers with high demands of care have less time to participate in such activities and rate their religiousness lower [43].

Intriguingly, in our analyses such characteristics of the carer as age, gender and education are not associated with the outcomes of family care. This differs from other research that suggests that gender and education in particular have an impact on carers’ perceived burden and service use, the latter being a support for family care [44,45]. We assume that other variables included in our analyses might overlay the effects of such factors on the outcomes of family care. In a similar way, one would have anticipated that our measures of the negative impact and positive value of care would have reached significance in our models. However, it may be that such generic measures of the caregiving situation, while valuable for describing how a carer experiences her or his role and for tailoring supportive interventions, may be less predictive of the outcomes of family care than the carer/care-recipient relationship and functional, cognitive and behavioural problems in the care-recipient.

This study has some limitations. One limitation may be the exclusion of the working conditions of the carers. It is known that the reconciliation of work and care is often a stressor for family carers, leading to a reduction in care hours provided or to giving up the caring role entirely, making the institutionalisation of care-recipients more likely [46,47]. Women especially, mainly caring daughters or daughters-in-law, are known to be an important source of informal care for older people and hence affected by the impact of care on their employment status [48]. However, one aim of this study was to give a picture of the associations between the carer/care-recipient and the outcomes of family care. For caring husbands, wives and partners, there is a statistical confound between the carer/care-recipient relationship and the working status of carer, as most of the carers are already retired. Thus, working conditions were excluded from the analyses.

Another limitation may be passage of time since the study data was collected. We argue that this limitation only marginally affects our fixed effects analyses, as care relationships and such characteristics of the care-recipient as functional, cognitive and behavioural problems are constant aspects of the family care of older people. However, country differences may have changed over the course of recent years due to structural changes in health care systems. The resourcing of health and social care, and so the amount of variance in our models explained at the country level, and the pattern of that variance as expressed by country, should be considered with caution.

Furthermore, we have high attrition rates for the follow-up sample in some countries. In order to assess the quality and representativeness of the follow-up data compared to the baseline data, national samples were compared for baseline and follow-up according to key characteristics, including demographic factors. Smaller deviations were found in the German and Greek follow-up samples, where the proportion of carers of more severely dependent older people is higher in the follow-up than in the baseline sample. For the UK, the divergence from baseline was slightly higher [49]. We applied response propensity weighting to account for response bias, especially between countries. Comparing the propensity scores between respondents and non-respondents (see supporting information S1 Fig) suggests that bias due to sample attrition is low.

Country findings have to be interpreted with caution. Although we have evidence on how different health care policies impact the situation of family care [39,40,50], there might be further indicators that are associated with a change of status in family care, like moral obligations, which are constituted by societies’ moral concepts and could only partially be reflected in our data.

Some of our measures, like care-recipients dependency or behavioural problems, are subjective assessments of the family carer and have to be interpreted with caution. However, the association between these factors and change of status obtained in our study and are in line with the findings of other studies.

Finally, where our carer participants were no longer providing care at follow-up, no interview was performed; it was felt inappropriate on ethical grounds to ask retrospectively about, for example, the negative impact or positive value of care, while the reliability of the data obtained would have been questionable. Hence, we could not analyse the effect of change in predictors over time on the outcomes of family care as addressed in this study.

## Conclusions

Family care of older adults is an important part of health care provision, not only for the quality of life of older adults who can “age in place” [51,52], but also regarding the economic value of family care. Supporting family care should be of great interest to policy makers. Our study shows that that the majority of family carers in our sample continued to provide care to their respective older relatives over a one-year period, despite often high levels of functional, cognitive and behavioural problems in the care-recipient and often when providing a high number of hours of care per week. Those family carers providing care to vulnerable care-recipients that are highly dependent, have behavioural problems and/or have dementia could benefit most from targeted and appropriate support. If the objective of health and social care services is to help the family carer to maintain their caregiving role, then our study suggests that support should be particularly guided toward those care dyads where there is no partners or spouse acting as carer, or no extended family network that might absorb the caring role when required. Distant relatives, friends or acquaintances who are acting as carers might need substantial intervention if their caregiving role is to be maintained. The maintenance of family care urgently requires a flexible structure, with community services and low-threshold services playing an important role in supporting carers and care-recipients [53].

Despite the longitudinal setting of this study, further research regarding the long-term impact of family care is still needed. The role of professional help and its effect on supporting the informal carer system over a longer period needs further investigation in particular, to see how support services can support both the family care situation and the well-being of carer and care-recipient.

## Supporting information

S1 Dataset EUROFAMCARE dataset used for the analyses, as RData file (to use with R statistics). (ZIP)

S1 Fig Distribution of adjusted inverse probability weights. (PDF)
