# Supplementary material for: For better or worse: Factors predicting outcomes of family care of older people over a one-year period. A six-country European study
Source: PLoS One. 2018 Apr 3;13(4):e0195294. doi: 10.1371/journal.pone.0195294 (PMC5882153; doi:10.1371/journal.pone.0195294)

Distribution of adjusted inverse probability weights

Comparison of the adjusted inverse probability weights for respondents (green) and non−respondents (red). Patterns indicate that our follow−up sample is rather a 'representative' subset of the baseline data.

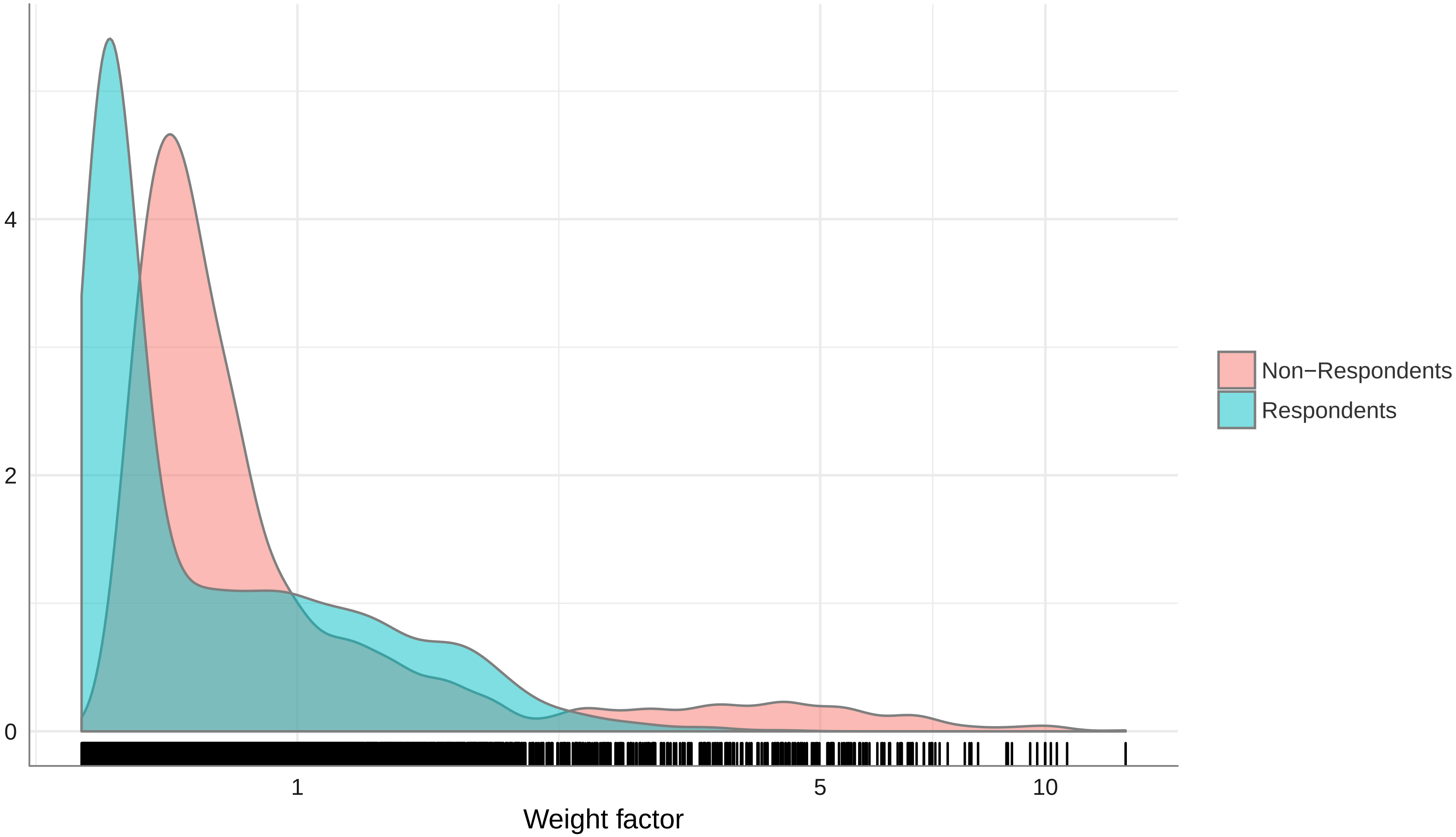

Supplement: S1 Fig — (PDF) [file pone.0195294.s002.pdf]
